# Post-transplant multimorbidity index and quality of life in patients with chronic graft-versus-host disease—results from a joint evaluation of a prospective German multicenter validation trial and a cohort from the National Institutes of Health

**DOI:** 10.1038/s41409-020-01017-8

**Published:** 2020-07-31

**Authors:** Daniel Wolff, Philipp Y. Herzberg, Anne Herrmann, Steven Z. Pavletic, Pia Heussner, Friederike Mumm, Christina Höfer, Inken Hilgendorf, Philipp G. Hemmati, Ernst Holler, Hildegard Greinix, Sandra A. Mitchell

**Affiliations:** 1 Department of Internal Medicine III, University Hospital of Regensburg, Regensburg, Germany; 2 Faculty of Humanities and Social Sciences, Personality Psychology and Psychological Assessment, Helmut Schmidt University of the Federal Armed Forces Hamburg, Hamburg, Germany; 3 Immune Deficiency Cellular Therapy Program, Center of Cancer Research, National Cancer Institute, Bethesda, MD USA; 4 Department of Medicine III, University Hospital, LMU Munich, Munich, Germany; 5 Klinik für Innere Medizin II, Abteilung für Hämatologie und Onkologie, Universitätsklinikum Jena, Jena, Germany; 6 Department of Hematology, Oncology and Tumor Immunology, Campus Virchow Klinikum Charité—University Hospital Berlin, Berlin, Germany; 7 Division of Hematology, Medical University of Graz, Graz, Austria; 8 Outcomes Research Branch, National Institutes of Health, Bethesda, MD USA

**Keywords:** Quality of life, Risk factors

## Abstract

Comorbidity after allogeneic hematopoietic stem cell transplantation (alloHSCT) impairs quality of life (QoL), physical functioning, and survival. We developed a new standardized measure to capture comorbidity after transplantation, the Post-transplant Multimorbidity Index (PTMI) in a cohort of 50 long term survivors. We subsequently evaluated the content validity and impact on survival and QoL within a multicenter trial, including 208 patients (pts) after alloHSCT, who were prospectively evaluated applying the FACT-BMT, the Human Activity Profile (HAP), the SF-36 v.2, PTMI and the Hematopoietic Cell Transplantation-Comorbidity Index (HCT-CI). The most prevalent comorbidities were compensated arterial hypertension (28.4%), ambulatory infections (25.5%), iron overload (23%), mild renal function impairment (20%), and osteoporosis (13%). Applying the PTMI 13% of patients had no comorbidity, while 37.1% had 1–3 comorbidities, 27.4% had 4–6 comorbidities, and 13.5% had > 6 comorbidities. Chronic graft-versus-host disease (cGvHD) was significantly associated with the PTMI, while age and prior acute GvHD were not. In contrast, the HCT-CI was not associated with the presence of cGvHD. cGvHD was significantly associated with depression (*r* = 0.16), neurological disease (*r* = 0.21), osteoporosis (*r* = 0.18) and nonmelanoma skin cancer (*r* = 0.26). The PTMI demonstrated strong measurement properties and compared to the HCT-CI captured a wider range of comorbidities associated with cGvHD.

## Introduction

While transplant related mortality (TRM) after allogeneic hematopoietic stem cell transplantation (alloHSCT) significantly decreased during the past 25 years [1], long term morbidity and late mortality remain a challenge [1–3]. Chronic graft-versus-host disease (cGvHD) is a direct risk factor for late mortality [4] and it induces subsequent comorbidity due to irreversible organ damage and toxicity of the immunosuppressive treatment [5, 6] resulting in a significantly higher prevalence of comorbidities in patients experiencing cGvHD [6–9]. Moreover, due to the development of toxicity-reduced conditioning regimens patients with higher comorbidity burden at the time of transplant and advanced age represent now a significant proportion of transplant survivors [10]. Also, the toxicity of the cytostatic treatment for malignant diseases before transplantation and during the conditioning regimen results in an increased incidence of secondary malignancies with cGvHD being a relevant additional factor [11–13]. Risk factors of cGvHD like low platelets have been shown to modulate the impact of comorbidities on mortality [14].

While the hematopoietic cell transplantation-specific comorbidity index (HCT-CI) has been primarily developed to predict pretransplant comorbidity related early TRM and has been validated in several cohorts, it also predicts late mortality in long term survivors developing cGvHD [15] but does not capture comorbidities potentially interfering with quality of life (QoL) and physical functioning (PF) during long term follow up after transplantation [14, 16–20]. Other tools for standardized comorbidity assessment like the Charlson Comorbidity Index (CCI) [21] and the Functional Comorbidity Index (FCI) [22] capture comorbidities interfering with QoL and PF but do not contain a number of comorbidities like infectious complications frequently present after alloHSCT. Acknowledging the critical impact of comorbidities on the interpretation of outcomes, the HCT-CI has become routine in reporting comorbidities of alloHSCT patients. Despite the high prevalence of comorbidities, in trials of new therapies for cGvHD, comorbid conditions are rarely reported, are not reported in a structured format that employs common definitions, and are not included in adjusted analyses of outcomes, although QoL and PF are regarded as valuable secondary endpoints [23]. Therefore, we developed and comparatively evaluated a new measure of comorbidity in long-term transplant survivors, the Post-transplant Multimorbidity Index (PTMI) [24], within a retrospective cohort of patients with cGvHD treated at the National Institutes of Health (NIH). We subsequently evaluated the performance of this new measure in a prospective multicenter trial validating the NIH consensus criteria for severity assessment of cGvHD. A secondary aim of this analysis was to explore the associations between the PTMI and HRQL, PF and late mortality. We also sought to describe the prevalence of comorbidities in long term survivors after alloHSCT. Moreover, we examined the association between cGvHD and comorbidity and prognosis. Finally, we compared the PTMI with established comorbidity measures including the HCT-CI, the CCI, and the FCI, respectively.

## Patients and methods

### Methods

#### PTMI

The PTMI was developed by a comprehensive analysis of reported comorbidities after alloHSCT and included all comorbidities with reported impact on survival, QoL or PF or/and were present within the development cohort excluding comorbidities which occurred randomly, were not directly or indirectly associated with the transplantation, and not considered to have an impact on any of the endpoints and did not require intervention like strabismus [25, 26].

The authors specified the following working definition of a comorbidity as a co-existing medical condition that is: (i) active (i.e. documented by radiographic, diagnostic testing, or laboratory evidence, or requiring either periodic surveillance/evaluation or medical management, or both); (ii) not a direct manifestation of cGvHD as defined by Filipovich et al. [27] and updated by Jagasia et al. [28], and (iii) not immediately resolved by prompt medical management. Based on the source materials, the PTMI was developed to reflect a broad and inclusive list of comorbid conditions that may arise in the post-transplant setting. To enhance longitudinal comparisons, the conditions and definitions captured by the HCT-CI [19], the CCI [21], and FCI [22] were included into the PTMI including the original definitions. The definitions for infectious comorbidities including severity grading were derived from previous work of Cordonnier et al. [29]. Comorbidities frequently present in patients after alloHSCT like hypo- and hyperthyroidism [30, 31], iron overload [32], arterial hypertension [33], pulmonary hypertension [34], growth failure and adrenal insufficiency due to the high frequency of long term exposure to corticosteroids [35] were also included. Mild renal insufficiency was added to the existing “moderate/severe renal disease” due the potential impact on treatment decision and the high frequency of renal impairment in alloHSCT survivors [36, 37]. Proteinuria was also included since most comorbidity indices capture renal impairment as defined by creatinine level, which often do not correlate with significant proteinuria. The relatively high threshold for proteinuria was chosen since proteinuria <1 g/day may temporarily occur even in benign orthostatic proteinuria [38]. Preliminary definitions to establish the presence of each of these conditions were either derived from the definitions employed by the commonly used comorbidity measures [19, 21, 22] or from case definitions presented in the literature for the diagnosis of specific conditions such as osteoporosis [39]. The conditions for which definitions were lacking (e.g. Post-transplant lymphoproliferative disease [40]) were developed by a consensus of transplant experts (SM, DW, EH, HG, BB, SZP), and iteratively refined to establish easy to apply definitions with low interrater variability. The definition for diabetes was added with a category “hyperglycemia” since steroids may result in a temporary hyperglycemia but often is not treated as classic manifest diabetes but nevertheless may influence treatment decisions and affect outcomes.

cGvHD-related manifestations were not defined as comorbid conditions. Accordingly, obstructive lung involvement due to cGvHD was excluded from the definitions of mild and moderate lung impairment and asthma within the PTMI; similarely liver involvement attributable to cGvHD was excluded from the definitions of mild or moderate hepatic impairment. Pulmonary hypertension was included since it potentially interferes with GvHD assessment. Since the PTMI applied the original definitions derived from already validated comorbidity tools (HCT-CI, CCI, and FCI) or international consensus definitions an analysis of the interrater—variability was only performed within the development cohort showing a very low variability.

The PTMI was calculated by summing up all present comorbidities with a score value of one for each comorbidity. Comorbidities with distinct severity categories were counted once only. Due to the limited number of patients included in both cohorts we did not perform analysis applying weighted comorbidities like the HCT-CI.

### Development cohort

To evaluate the content validity of the PTMI, the latter was comparatively evaluated in a cohort of 50 alloHSCT survivors referred from allogeneic transplant centers around the United States to the NIH for comprehensive cGvHD consultation. Data were obtained from a natural history study prospectively examining the course of cGvHD (NCT00092235). Participants were eligible to participate if they were: (1) aged ≥ 18, (2) able to speak, read and write English, (3) at least 100 days post alloHSCT from a matched related or unrelated donor, and (4) carried a diagnosis of cGvHD established through clinical signs and/or tissue biopsy of one or more organ systems. Clinical and illness-related data including comorbidities were gathered through a comprehensive history and physical with a physician or nurse practitioner, and through a series of diagnostic and laboratory evaluations. All participants provided written informed consent to participate in the study. Patient characteristics are shown in Table 1.

**Table 1:** Patient characteristics.

No. of patients	50 (Development)	208 (Validation)
	*n* (%)	*n* (%)
GvHD characteristics		
No evidence of GvHD	1 (2)	72 (34.6)
Late acute GvHD	1 (2)	0
Classic cGvHD	48 (96)	136 (65.4)
NIH severity: mild	4 (8)	36 (17.3)
Moderate	8 (16)	62 (29.8)
Severe	36 (72)	38 (18.3)
Distribution of organ manifestations of cGvHD	*n* = 48	
Cutaneous	40 (83.3)	113 (83.1)
Oral	22 (45.8)	96 (70.6)
Eye	48 (100)	89 (65.4)
Gastrointestinal	3 (6)	48 (35.3)
Liver	8 (16.7)	60 (44.1)
Lung	12 (25)	30 (22.1)
Fascia	13 (27)	35 (25.7)
Median Age (range) in years	43 (20–69)	44 (18–72)
Gender (male/female) %	62/38	51/49
Graft source		
PBSC	40 (80)	183 (88)
BM	10 (20)	18 (9)
Other	0	7 (3)
Median days from alloHSCT to evaluation (range)	1216 (390–6240)	272 (85–4003)
Median days from alloHSCT to onset of cGvHD	240 (120–244)	200 (92–2030)

*No* number, *PBSC* peripheral blood stem cells, *BM* bone marrow, *alloHSCT* allogeneic hematopoietic stem cell transplantation.

### Validation cohort

Subsequently, the PTMI was evaluated within a prospective multi-center study evaluating the NIH cGvHD consensus recommended data collection instruments including the FACT-BMT [41], human activity profile (HAP) [42], the SF36 [43], and the Lee-cGVHD-Symptom-Scale (L-cGVHD-SC) [23, 27, 28, 42, 44, 45]. The Hospital Anxiety and Depression Scale (HADS) was added to capture the latter symptoms [46]. The comorbidities arterial hypertension, renal insufficiency, hyperglycemia, diabetes, osteoporosis, osteonecrosis, cataract, hypothyroidism, infections, polyneuropathy, and venous thrombosis were captured within the original documentation form applying standard criteria. In all other comorbidities captured by the PTMI the missing information was collected retrospectively by chart review evaluating results of routine follow up examinations according to the EBMT guidelines [47]. The study was reviewed and approved by an Institutional Review Board at each participating center. Patients were enrolled during regular visits at the transplant center after providing signed informed consent between day 100 and 1 year after alloHSCT, or in the presence of active cGvHD without time limit. Exclusion criteria were life expectancy <3 months, inability to understand and fill out the forms without assistance, or relapse of the underlying malignancy. Two-hundred eight patients who received an alloHSCT for hematologic malignancies (*n* = 206), aplastic anemia (*n* = 1), or paroxysmal nocturnal hemoglobinuria (*n* = 1) were included at six German and one Austrian institutions. Patient characteristics are shown in Table 1.

### Statistical analysis

Descriptive characteristics of the sample are reported. To investigate the association of the comorbidity indices PTMI, FCI, CCI, and HCT-CI with single comorbidities (e.g. hypogonadism), Spearman’s rank correlation coefficients were calculated. In terms of the association of cGvHD with specific comorbidities, Pearson correlation coefficients were calculated. The associations of the PTMI, FCI, CCI and HCT-CI with QoL and PF outcomes were investigated calculating Spearman-Rho correlation coefficients. To identify predictor variables of the specific comorbidity indices (HCT-CI, FCI, CCI, and PTMI), stepwise regression analyses were conducted, for which underlying assumptions were judged satisfactory. *p* < 0.05 was considered statistically significant. For survival analysis, Kaplan–Meier curves were computed. All analyses were carried out using SPSS 22.0 for Windows.

## Results

### Comorbidities

Development cohort: Using the PTMI, only 1 patient (2%) had no comorbidity, while 10 (20%) had 1–3 comorbidities, 25 (50%) 4–6 comorbidities, and 14 (28%) >6 comorbidities. Applying the four comorbidity indices in our development cohort of 50 subjects with cGvHD, we found a mean of 5.2 (SD ± 2.3) (PTMI), 1.5 (SD ± 1.23) (HCT-CI), 1.39 (SD ± 0.78) (CCS), and 2.18 (SD ± 1.16) (FCI) co-occurring conditions. On average the FCI missed the identification of one or more comorbidities 43% of the time, CCS 75% of the time, and HCT-CI 71% of the time. Conditions that were highly prevalent in the development cohort but missed by the other comorbidity measures included osteoporosis (75%), avascular necrosis (18%), hypertriglyceridemia (22%), hypothyroidism (25%), BMI < 22 (42%), and secondary solid malignancy (excluding nonmelanoma skin cancer) after transplant (10%). Using the PTMI, the two most prevalent comorbid conditions were osteoporosis (75%) and underweight/sarcopenia (BMI < 22) (42%), whereas the FCI identified osteoporosis and depression as most prevalent, the HCT-CI identified psychiatric disturbance and peptic ulcer, and the CCS identified history of malignancy and peptic ulcer disease, as the two most prevalent comorbidities, respectively.

An association between the total number of PTMI-defined comorbid conditions and time since cGvHD diagnosis was seen. Specifically, study participants who were 40 months or more after transplant had significantly more PTMI-defined comorbid conditions (6 conditions versus 4; *p* < 0.03), compared to those who were less than 40 months post-transplant. There was no association between the median number of PTMI-defined comorbidities and age (*p* = 0.12).

Validation cohort: According to the PTMI, 27 (13%) patients had no comorbidity, while 77 (37%) had 1–3 comorbidities, 57 (27%) 4–6 comorbidities, and 28 (13.5%) >6 comorbidities. In contrast to the PTMI, the HTC-CI only classified 6 (2.9%) of the patients as multimorbid with >6 comorbidities, respectively. The frequency of individual comorbidities is shown in Table 2 and the distribution of the number of captured comorbid conditions in relationship to the assessment tool applied is listed in Table 3.

**Table 2:** Frequency of comorbidities captured by the PTMI including the applied definition. Comorbidities and their definitions as captured by the HCT-CI, FCI, CCI that are also nested within the PTMI are indicated.

Comorbidity	Definition	Development cohort (%) *n* = 50	Present (%) *n* = 207	Present in cGvHD patients (%) *n* = 136	Present in patients without cGvHD (%) *n* = 68
Cardiovascular					
Arrhythmia (HCT-CI, FCI)	Atrial fibrillation or flutter, sick sinus syndrome or ventricular arrhythmias	2	1.9	1.5	2.8
Coronary artery disease (HCT-CI, FCI)	Coronary artery disease (one or more vessel-coronary artery stenosis requiring medical treatment, stent, or bypass graft)	2	2.9	3.7	1.4
History of MI (HCT-CI, FCI, CCI)	History of myocardial infarction	2	0.5	0.7	none
Heart valve disease (HCT-CI, FCI)	Except asymptomatic mitral valve prolapse and trace/physiologic regurgitation on echocardiogram	none	9.6	8.8	11.3
Congestive heart failure (FCI, HCT-CI, CCI)					
Mild/moderate	EF 30–50%	6	1.9	2.2	1.4
Severe	EF < 30%	none	none	none	none
Cerebrovascular disease (HCT-CI, FCI, CCI)	Transient ischemic attack or history of cerebrovascular accident, or neurologic impairment consequent to CVA	none	1	0.7	none
Peripheral vascular disease (FCI, CCI)	Resulting in impairment of daily activities and requiring pharmacologic treatment	none	1	0.7	1.4
Venous thrombosis	Confirmed by radiographic/ultrasound testing and requiring anticoagulation	6	9.6	10.3	8.5
Arterial hypertension					
Compensated arterial hypertension	Receiving an antihypertensive agent for treatment of hypertension	30	28.4	25.7	33.8
Uncontrolled arterial hypertension	Repeated blood pressure measurements greater than 160 systolic or greater than 100 diastolic, despite pharmacologic intervention	6	none	none	none
Gastrointestinal					
Peptic ulcer/hernia/reflux (HCT-CI, FCI, CCI)	Requiring treatment	40	1.4	2.2	none
Mild hepatic (HCT-CI)	Chronic hepatitis, bilirubin >ULN to 1.5 x ULN, or AST/ALT > ULN to 2.5 x ULN	8	20.2	22.8	14.1
Moderate/severe hepatic (HCT-CI, CCI)	Liver cirrhosis, bilirubin >1.5 times ULN or AST/ALT > 2.5 x ULN not attributable to cGvHD	2	7.7	9.6	4.2
Iron overload	Ferritin > 1000 µg/ml	2	23	24.3	21.1
Hepatitis B	Hep B surface-antigen positive and/or PCR positive or receiving treatment for Hep B	None	2.4	3.7	none
Hepatitis C	PCR positive or receiving treatment for Hep C	none	none	none	none
Inflammatory bowel disease (HCT-CI)	Crohn’s disease or ulcerative colitis prior to transplant	none	0.5	none	1.4
Pulmonary					
Moderate pulmonary (HCT-CI, FCI, CCI)	DLCO and/or FEV-1 66%-80% with dyspnea on slight activity not attributable to cGvHD	5	13.5	16.2	8.5
Severe pulmonary (HCT-CI, FCI, CCI)	DLCO and/or FEV-1 65% or lower with dyspnea at rest or requiring oxygen not attributable to cGvHD	5	7.7	11.0	1.4
Pulmonary hypertension	Mean pulmonary artery pressure in rest >25 mm Hg or systolic pulmonary artery pressure at rest >40 mmHg	none	2.4	1.5	4.2
Asthma (FCI)	Asthma symptoms for which inhaled steroids or other daily treatments are needed chronically to prevent or manage attacks and/or reversible obstruction on pulmonary function tests	none	0.5	0.8	none
Endocrine					
Hyperglycemia	Fasting blood sugar greater than ≥126 mg/dl or random blood sugar greater than 200 mg/dl	none	2.9	3.3	2.8
Diabetes (HCT-CI, FCI, CCI)	Requiring treatment with insulin or oral hypoglycemic agents but not diet alone	24	9.1	9.6	8.5
Hypothyroidism	Including compensated hypothyroidism	25	9.6	8.4	14.1
Hyperthyroidism	Suppressed TSH not associated with thyroid hormone over-replacement	none	1.9	3.3	none
Hypogonadism (postmenopausal, testosterone level)	Women who are post-menopausal below the age of 45 years (at least 6 months without menses), need for testosterone replacement or free testosterone below the lower limit	65	9.6*	13.2	2.9
Adrenal insufficiency	Including compensated adrenal insufficiency requiring substitution	4	2.9	2.2	4.4
Hypertriglyceridemia	Requiring treatment or above 2 times the upper normal limit	22	9.6	11.8	5.9
Hypercholesterolemia	Requiring treatment or total cholesterol greater than 1.5 times the upper limit of normal value or LDL cholesterol > 160 mg/dl	16	12	14.7	7.0
Bone/joint					
Osteoarthritis (FCI)	Symptomatic and requiring treatment, or symptomatic and with osteoarthritic changes noted on radiographic studies	None	None	None	None
Degenerative disc disease (spinal stenosis or severe chronic back pain) (FCI)	Symptomatic and requiring treatment or symptomatic and with degenerative disc disease noted on radiographic studies	10	3.8	4.4	2.8
Avascular necrosis	Symptomatic with pain secondary to AVN or joint replacement	18	2.4	3.7	none
Osteopenia/Osteoporosis (FCI)	T Score < or equal to minus 1.5 or on treatment with a bisphosphonate (not as part of treatment for MM)	75	13	18.4	2.8
Rheumatologic (HCT-CI, FCI, CCI)	Lupus, mixed connective tissue disorder, rheumatoid arthritis (confirmed serological and/or by typical Xray), polymyalgia rheumatic	None	1	1.5	none
Neuropsychiatric					
Psychiatric disturbance-Depression (HCT-CI, FCI)	Depression requiring psychiatric consult or treatment for depression	32	8.2	8.1	8.5
Psychiatric disturbance-Anxiety or panic disorder (HCT-CI, FCI)	Anxiety or panic disorder requiring psychiatric consult or treatment for anxiety	8	8.2	6.6	11.3
Dementia (CCI)	Presence of neurocognitive impairment including memory, attention, language, and problem solving persisting for at least 6 months	None	None	none	none
Neurologic disease (peripheral neuropathy, MS, Parkinson’s disease or other chronic neurologic disease) (FCI, CCI)	Symptomatic and requiring treatment to control or manage symptoms/disease process	30	9.1	9.6	8.5
Visual impairment secondary to cataracts, glaucoma or macular degeneration (FCI)	Unilateral or bilateral, and unrepaired	32	4.3	5.1	2.8
Hearing impairment (FCI)	Very hard of hearing, even with hearing aids	None	1.4	2.2	none
Other comorbidities					
Infection: (PTMI only)	Requiring treatment with an antimicrobial drug (not prophylaxis) within the past three months (count only one infection during past three months, and score for most severe infection)				
Mild infection	Any mild infection which has been treated as outpatient or did not require hospitalization for itself (oral HSV or candida), oral treatment for bacterial infection (without pneumonia), asymptomatic CMV reactivation	22	25.5	26.5	23.9
Moderate infection	Infection requiring hospitalization for supportive care/treatment, or intravenous antibiotics or CMV/VZV with fever only, or fungal pneumonia without respiratory/constitutional symptoms.	None	11.1	8.1	16.9
Severe infection	Admitted for treatment and with life threatening manifestations/consequences of infection (eg. severe sepsis, organ complications, aspergillosis- associated organ destruction/invasion, viral encephalitis, severe varicella zoster with organ complications such as pneumonia, zoster ophthalmicus, hepatitis, CMV with enteritis, hepatitis, or pneumonia; PCP infection with respiratory symptoms, toxoplasmosis with organ involvement, nocardia infection)	6	1.9	2.9	none
Mild renal	Serum creatinine>1.2 mg/dl but less than 2 mg/dl	4	20	17.6	25.4
Moderate/severe renal (HCT-CI, CCI)	Serum creatinine≥2 mg/dl, on dialysis, or prior renal transplantation	None	1.4	0.7	2.8
Proteinuria	>1 gram/day or 1 g/g creatinine	4	1.4	2.2	none
Prior solid malignancy (HCT-CI, CCI)	Treated at any time point in the patient’s past history before transplantation, excluding nonmelanoma skin cancer	8	3.8	2.2	7.0
Solid malignancy (CCI)	Occurrence of a solid malignancy (excluding nonmelanoma skin cancer) after transplantation	10	None	none	none
Nonmelanoma skin cancer (CCI)	Occurrence of a non-melanoma skin cancer after transplantation	14	1.9	2.9	none
PTLD (CCI)	Requiring intervention (reduction or withdrawal of immunosuppression or treatment with rituximab or adoptive cellular treatment	2	None	none	none
Obesity (FCI)	Body mass index >30 and <35 (weight in kg/height in meters)	12	3.4	4.4	1.4
Obesity (HCT-CI, FCI)	Obesity and/or body mass index ≥35 (weight in kg/height in meters)	None	1	none	2.8
Underweight	Body mass index<18.5	42	7.7	8.8	5.6
Secondary growth failure	Speed of growth below 25^th^ percentile	None	None	none	none
Nicotine abuse	Daily use/consumption of any substance containing nicotine	4	4.3	4.4	4.2
PTMI Sum Score (Mean/SD/Range)	Sum number of comorbid conditions	5.2 (±2.3) Range 0–11)	3.6 (0–15)	3.97** (0–15)	3.0 (0–10)

Comorbidities and their definitions as captured by the HCT-CI, FCI, CCI that are also nested within the PTMI are indicated.

*Applying the definition of hypogonadism as being postmenopausal <45 years 18.3% of females had hypogonadism.

**Differences between the PTMI score of patients with cGvHD versus without cGvHD were significant (*p* <  0.05).

**Table 3:** Comparison of the number of concurrent comorbid conditions captured by each of the four measures in the validation cohort.

	PTMI		HCT-CI		CCI		FCI	
No. of comorbidities	Frequency	%	Frequency	%	Frequency	%	Frequency	%
0	27	13.0	47	22.6	106	51.0	88	42.3
1	18	8.7	40	19.2	25	12.0	53	25.5
2	27	13.0	24	11.5	12	5.8	22	10.6
3	32	15.4	19	9.1	21	10.1	15	7.2
4	31	14.9	29	13.9	6	2.9	3	1.4
5	20	9.6	13	6.3	4	1.9	6	2.9
6	6	2.9	11	5.3	3	1.4	1	0.5
7	10	4.8	2	1	3	1.4	1	0.5
8	5	2.4	0	0	6	2.9		
9	5	2.4	1	0.5	0	0		
10	2	1.0	2	1.9	2	1.0		
11	1	0.5	1	0.5	0	0		
12	0	0			1	0.5		
13	3	1.4						
14	1	0.5						
15	1	0.5						

**Fig. 1:**
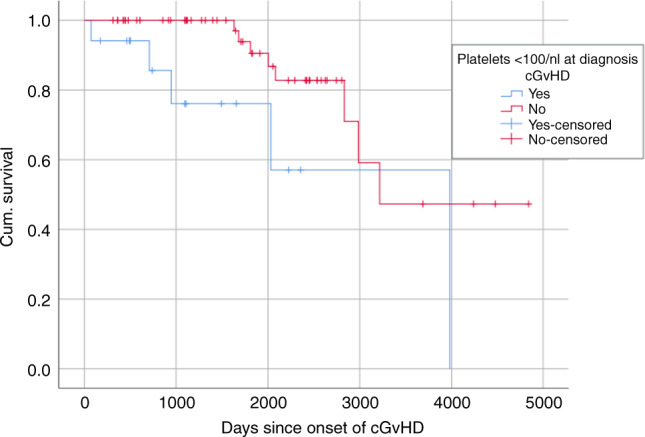
Impact of platelets at diagnosis of cGvHD on survival. The red (upper) Kaplan-Meier survival curve indicates platelets > 100/nl, the blue (lower) curve platelets < 100/nl. The differences were significant (*p*=0.025) applying the log rank test.

The results on the association of QoL with single comorbidities (Table 4), the association of the PTMI, FCI, CCI and HCT-CI with QoL and PF (Table 5), as well as the impact of cGvHD on comorbidities and survival (Fig. 1) are provided within the supplemental material.

**Table 4:** Association of Quality of Life outcomes with single comorbidities.

	FACT physical well-being	FACT social well-being	FACT emotional well-being	FACT functional well-being	FACT total	SF36 physical functioning	SF36 role physical	SF38 bodily pain	SF36 general health	SF36 vitality	SF36 role emotional	SF36 mental health	HAP MAS	HAP AAS	HAP HCT-adjusted AAS	HADS anxiety	HADS depression
Compensated hypertension	*r* = −0.15 *p* = 0.014		*r* = −0.16 *p* = 0.029			*r* = −0.22 *p* = 0.003	*r* = −0.15 *p* = 0.047			*r* = −0.18 *p* = 0.016	*r* = −0.25 *p* = 0.001	*r* = −0.16 *p* = 0.030	*r* = −0.19 *p* = 0.008	*r* = −0.22, *p* = 0.002	*r* = −0.23, *p* = 0.002		*r* = 0.16 *p* = 0.028
Hypogonadism	*r* = −0.24 *p* = 0.001	*r* = −0.26 *p* < 0.001	*r* = −0.24 *p* = 0.001	*r* = −0.24 *p* = 0.001	*r* = −0.30 *p* < 0.001	*r* = −0.24 *p* = 0.001			*r* = −0.18 *p* = 0.014	*r* = −0.20 *p* = 0.008		*r* = −0.20 *p* = 0.008			*r* = −0.15, *p* = 0.045	*r* = 0.20,*p* = 0.008	*r* = 0.27 *p* < 0.001
Severe infection	*r* = −0.15 *p* = 0.040			*r* = −0.16 *p* = 0.032													*r* = 0.15 *p* = 0.040
Moderate infection						*r* = −0.16 *p* = 0.025							*r* = −0.18 *p* = 0.013	*r* = −0.23 *p* = 0.001	*r* = −0.22 *p* = 0.002		
Mild infection		*r* = −0.22 *p* = 0.002	*r* = −0.16 *p* = 0.025									*r* = −0.17 *p* = 0.020					
Hepatitis B			*r* = −0.22 *p* = 0.003		*r* = −0.16 *p* = 0.031				*r* = −0.19 *p* = 0.009								
Venous thrombosis				*r* = −0.18 *p* = 0.014	*r* = −0.16 *p* = 0.031	*r* = −0.20 *p* = 0.006	*r* = −0.15 *p* = 0.038		*r* = −0.15 *p* = 0.042				*r* = −0.16 *p* = 0.025	*r* = −0.20 *p* = 0.005	*r* = −0.19 *p* = 0.011		
Mild renal							*r* = −0.15 *p* = 0.043		*r* = −0.19 *p* = 0.011		*r* = −0.18 *p* = 0.013						
Iron overload								*r* = 0.18 *p* = 0.022									
Proteinuria								*r* = 0.18 *p* = 0.022		*r* = −0.19 *p* = 0.024							*r* = 0.15 *p* = 0.037
Hyper-cholesterinemia										*r* = −0.18 *p* = 0.014							
Pulmonary hypertension																	*r* = 0.16 *p* = 0.039

**Table 5:** Spearman-Rho correlations between the PTMI, FCI, HCT-CT, and CCI with QoL outcomes.

	PTMI	FCI	HCT-CI	CCI
FACT-BMT physical well-being	−0.25**	−0.28***	−0.23**	−0.25***
FACT-BMT social/family well-being	−0.26***	−0.31***	−0.19**	−0.14
FACT-BMT emotional well-being	−0.24**	−0.31***	−0.16*	−0.15*
FACT-BMT functional well-being	−0.26***	−0.27***	−0.18*	−0.20**
FACT-BMT total Score	−0.29***	−0.35***	−0.22**	−0.21**
SF-36 physical functioning (PF)	−0.34***	−0.34***	−0.24**	−0.23**
SF-36 role-physical (RF)	−0.15*	−0.11	−0.12	−0.11
SF-36 bodily pain (BP)	−0.11	−0.20**	−0.12	−0.14*
SF-36 general health (GH)	−0.30***	−0.31***	−0.18*	−0.19*
SF-36 vitality (VT)	−0.25***	−0.24***	−0.22*	−0.19**
SF-36 social functioning (SF)	−0.16*	−0.15*	−0.12	−0.06
SF-36 role-emotional (RE)	−0.13	−0.12	−0.04	−0.02
SF-36 mental health (MH)	−0.27***	−0.34***	−0.23**	−0.19**
HAP-MAS	−0.16*	−0.19**	−0.14	−0.16*
HAP-AAS	−0.24***	−0.22**	−0.19**	−0.21**
HAP-HSCT-AAS	−0.25***	−0.26***	−0.20**	−0.21**
HADS anxiety	0.21**	0.30***	0.15*	0.16*
HADS depression	0.26***	0.28***	0.17*	0.18*

*p < 0.05, **p ≤ 0.01,***p ≤ 0.001.

## Discussion

While survival after alloHSCT has improved significantly over the last 20 years [1], long term morbidity and mortality have remained important issues with cGvHD having the most impact [6, 48]. Although comorbidity burden remains a challenge after alloHSCT, until now, standardized documentation of comorbid long-term effects during post-transplant follow up has been lacking. Consequently, cGvHD trials have emphasized the toxicity directly associated with GvHD-directed interventions, rather than the comorbidity burden in the treated population [6, 12, 49–51].

The four comorbidity measures examined produced important differences in the average number of comorbid conditions identified, and the HCT-CI, CCS, and FCI did not identify one or more comorbidities 71%, 75%, and 43% of the time. Therefore, the choice of a comorbidity measure is an important methodologic consideration in the design of studies of comorbidity in post-transplant survivors, including those with cGvHD.

Comorbidities in cGvHD can also substantially complicate systemic therapy management, drug dosing and tolerability. While the HCT-CI has been primarily developed and validated to predict early post-transplant mortality [18–20, 52–54], it may also predict late mortality in patients with cGvHD [14, 15]. Nevertheless, comorbidities like osteoporosis frequently present in long term survivors and repeated infections after alloHSCT are not captured and consequently, HCT-CI score did not correlate with the presence and severity of cGvHD. In contrast, severity of cGvHD predicted the new PTMI sum score indicating a superior sensitivity to capture sequelae induced by cGvHD and its treatments. Moreover, the PTMI performed superior in predicting QoL and PF in comparison to the HCT-CI indicating, that the burden of concomitant diseases is of relevance for QoL and PF. Of interest, the PTMI classified a significantly higher proportion of patients as multimorbid (>6 comorbidities); 28% in the development and 13.5% of patients of the validation cohort) while none of those in the development cohort and only 3.9% of patients in the validation cohort were classified as multimorbid by the HCT-CI. Moreover, the CCI classified none of those in the development cohort and only 5.8% of patients as multimorbid reflecting the fact that the primary purpose of the CCI is to capture specific comorbidities potentially relevant for mortality outside the transplantation setting [21].

Leading single comorbidities, which were captured solely by the PTMI were mild renal impairment, infectious complications treated on an outpatient basis and osteoporosis which is in line with prior reports [55]. Mild renal impairment occurs frequently either as sequelae of immunosuppressive treatment or the conditioning regimen [36, 56, 57] while infectious complications have been observed mainly in association with cGvHD [58]. Of interest, a relative high frequency of neurological sequelae was detected indicating, that neurological problems are of significant relevance after alloHSCT either as the result of toxicity or as associated manifestation of cGvHD [59–61]. Despite lower frequency compared to the general population, nicotine abuse was reported indicating the need for behavioral intervention within this cohort already predisposed to higher risk for secondary malignancies and cardiovascular sequelae [62]. The high incidence of hypercholesterolemia was confirmed although the incidence of cardiovascular sequelae remained low which may have been due to the relatively short follow up compared to other reports [63, 64]. Not surprisingly, a high frequency of iron overload was noted [65]. With regard to hypogonadism which frequently occurs in female patients after alloHSCT [51, 66] the relatively low frequency reported may have been due to the lack of treatment of hypogonadism despite premature menopause and the mean patients’ age of 44 years.

Moreover, the higher frequency of depression in patients with cGvHD was confirmed indicating the need for psychological support of survivors coping with cGvHD [67–69]. The lack of impact of the comorbidity burden on survival may be partially explained by the study design which included either long term survivors with cGvHD or patients without GvHD during the 1st year after transplant and excluded patients with high risk for early mortality. Nevertheless, the presence of low platelet count as risk factor for mortality at diagnosis of cGvHD was confirmed [14, 70].

Several caveats should be considered in interpreting our findings. We acknowledge that it can be difficult to separate manifestations of cGvHD from comorbidity, particularly with respect to pulmonary, rheumatologic, and hepatic conditions. Though the definitions included in the PTMI isolate manifestations of cGvHD from comorbid complications of cGvHD treatment and other late post-transplant complications, additional studies are needed to examine the specificity and interrater reliability of the measure with respect to distinguishing cGvHD manifestations from comorbidity. The PTMI shares some of the limitations of comorbidity indices more generally, including the fact that such indices do not fully account for the severity or duration of the comorbid condition, although the PTMI attempts to distinguish compensated from uncompensated conditions. Comorbidity indices may also be sensitive to biases introduced by incomplete case ascertainment. However, a strength of this comparative analysis is the fact that in both cohorts, all participants were thoroughly characterized through comprehensive history and physical examination, as well as clinical, radiologic, and laboratory evaluation. Nevertheless, we can not exclude that within the validation cohort some comorbidities may have underdiagnosed due to the absence of diagnostic measures (i.e. osteoporosis may have been underdiagnosed to lack of bone density assessment). An additional limitation is the too low number of patients in the validation cohort to permit meaningful analysis weighting different comorbidities according to their individual impact on the endpoints taking into account that the individual weighting may be endpoint dependent. (The HCT-CI focused on treatment related mortality while QoL may influenced by other comorbidities.) Nevertheless, a strength is the analysis of two cohorts from different countries and broad diversity with regard to cGvHD status, time since transplant and age.

In summary, our findings support the content validity of the PTMI and suggest that this new comorbidity measure improves the identification of comorbid conditions in long-term transplant survivors. Of note, the PTMI demonstrated superior performance in detecting the impact of cGvHD and showed a higher correlation to QoL and PF variables compared to the HCT-CI indicating its utility, particularly in cGvHD therapy trials. Moreover, a broad variety of comorbidities are captured according to standardized definitions, thereby permitting comparisons of comorbidity prevalence. Our results may also have implications for trial conduct, including eligibility criteria, patient management, trial interpretation, pre-emptive management of drug-drug interactions and adverse event reporting. With continued testing and refinement including weighting of specific comorbidities, we anticipate that this new measure will have utility for risk-adjustment and stratification in observational studies and clinical trials in the post-transplant setting, permitting comparison of comorbidity burden across clinical trials.

## Supplementary information

Supplemetary Data
