# Correction: Post-transplant multimorbidity index and quality of life in patients with chronic graft-versus-host disease—results from a joint evaluation of a prospective German multicenter validation trial and a cohort from the National Institutes of Health

**DOI:** 10.1038/s41409-021-01404-9

**Published:** 2021-08-19

**Authors:** Daniel Wolff, Philipp Y. Herzberg, Anne Herrmann, Steven Z. Pavletic, Pia Heussner, Friederike Mumm, Christina Höfer, Inken Hilgendorf, Philipp G. Hemmati, Ernst Holler, Hildegard Greinix, Sandra A. Mitchell

**Affiliations:** 1 Department of Internal Medicine III, University Hospital of Regensburg, Regensburg, Germany; 2 Faculty of Humanities and Social Sciences, Personality Psychology and Psychological Assessment, Helmut Schmidt University of the Federal Armed Forces Hamburg, Hamburg, Germany; 3 Immune Deficiency Cellular Therapy Program, Center of Cancer Research, National Cancer Institute, Bethesda, MD USA; 4 Department of Medicine III, University Hospital, LMU Munich, Munich, Germany; 5 Klinik für Innere Medizin II, Abteilung für Hämatologie und Onkologie, Universitätsklinikum Jena, Jena, Germany; 6 Department of Hematology, Oncology and Tumor Immunology, Campus Virchow Klinikum Charité—University Hospital Berlin, Berlin, Germany; 7 Division of Hematology, Medical University of Graz, Graz, Austria; 8 Outcomes Research Branch, National Institutes of Health, Bethesda, MD USA

**Keywords:** Quality of life, Risk factors

Correction to: *Bone Marrow Transplantation* 10.1038/s41409-020-01017-8

The article “Post-transplant multimorbidity index and quality of life in patients with chronic graft-versus-host disease—results from a joint evaluation of a prospective German multicenter validation trial and a cohort from the National Institutes of Health”, written by Daniel Wolff et al., was originally published electronically on the publisher’s internet portal on 31 July 2020 without open access. With the author’ decision to opt for Open Choice the copyright of the article changed on 6 June 2021 to © Author(s) 2020 and the article is forthwith distributed under a Creative Commons Attribution 4.0 International License, which permits use, sharing, adaptation, distribution and reproduction in any medium or format, as long as you give appropriate credit to the original author(s) and the source, provide a link to the Creative Commons licence, and indicate if changes were made. The images or other third-party material in this article are included in the article’s Creative Commons licence, unless indicated otherwise in a credit line to the material. If material is not included in the article’s Creative Commons licence and your intended use is not permitted by statutory regulation or exceeds the permitted use, you will need to obtain permission directly from the copyright holder. To view a copy of this licence, visit http://creativecommons.org/licenses/by/4.0. Open access funding enabled and organized by Projekt DEAL.

