# Supplementary material for: Post-transplant multimorbidity index and quality of life in patients with chronic graft-versus-host disease—results from a joint evaluation of a prospective German multicenter validation trial and a cohort from the National Institutes of Health
Source: Bone Marrow Transplant. 2020 Jul 31;56(1):243–56. doi: 10.1038/s41409-020-01017-8 (PMC8376641; doi:10.1038/s41409-020-01017-8)
Supplement: Supplementary file 1 — Supplemetary Data [file 41409_2020_1017_MOESM1_ESM.docx]

**Association of Quality of Life outcomes with single comorbidities**

Hypogonadism was the comorbidity with a negative impact on 12 of the reported QoL scales. The association persisted when all female patients being postmenopausal below the age of 45 years were regarded as suffering from hypogonadism with significant association with the FACT social/family Well-Being (r= -.15, p= .034), FACT emotional Well-Being (r= -.17, p= .016), FACT functional Well-Being (r= -.16, p= .031) subscale and the FACT total score (r= -.18, p= .014. Furthermore, it is noteworthy, that compensated hypertension had an impact on 11 and venous thrombosis on eight of the reported scales. As shown in table 4 a significant number of additional reported comorbidities showed associations with QoL outcomes (FACT-BMT, SF-36, HAP and HADS). The PTMI was the only Comorbidity-Index that captured the reported comorbidities.

Since we excluded moderate and severe pulmonary problems including asthma and hepatic dysfunction in patients with cGvHD – they are regarded as manifestations of cGvHD - we additionally evaluated their impact on QoL and PF. While hepatic dysfunction had no impact on QoL and PF, moderate lung problems correlated with the FACT emotional (r= -.17, p= .023), FACT functional subscale (r= -.17, p= .020), and FACT total score (r= -.16, p= .026). In addition, moderate lung impairment correlated inversely with the SF36 mental health subscale (r= -.20, p= .007) and the HADS depression scale (r= -.19, p= .011). Severe lung problems correlated inversely with the FACT physical subscale (r= -.20, p= .007) and FACT total score (r= -.17, p= .018), as well as the SF36 physical (r= -.19, p= .012) and mental health subscale (r= -.18, p= .015). Moreover, severe lung involvement correlated with the HADS anxiety (r= -.19, p= .012) and HADS depression scale (r= -.19, p= .010).

**Association of the PTMI, FCI, CCI and HCT-CI with QoL and PF outcomes**

The PTMI, HCT-CI, FCI and CCI were significantly negatively associated with QoL outcomes (FACT-BMT, SF-36, HAP). The detailed results are presented in Table 5.

The PTMI, FCI and HCT-CI were significantly associated with all FACT-BMT subscales and the FACT-BMT total score. The CCI was associated with only three of the four FACT-BTM subscales (Physical Well-Being, Emotional Well-Being, Functional Well-Being) and the FACT-BMT total score. Referring to the SF-36, the PTMI was associated with six of the eight subscales, whereas the HCT-CI was only associated with four of the eight subscales. In contrast to the FCI, HCT-CI and the CCI, the PTMI was the only comorbidity index that was significantly associated with the SF-36 subscale ‘physical role function‘. All indices showed significant associations with the HADS Depression and Anxiety subscales and all HAP- subscales. The HCT-CI was the only index that was not associated with the HAP.

**Impact of cGvHD on comorbidities**

Since the NIH cohort included only one participant without cGvHD, analysis of the association between cGvHD and comorbidities was restricted to the validation cohort. Chronic GvHD was significantly positively correlated with hypogonadism (*r*= .19, *p*< .02), and osteopenia/osteoporosis *(r*= .25, *p*= .001). The association with cGvHD was no longer detectable, if all females being postmenopausal below the age of 45 years were regarded as having hypogonadism by definition. Of note, while metabolic bone diseases were the only single comorbidity with significant association with cGvHD, the latter was associated with a significant higher sum of comorbidities (mean 4 versus 2 in patients without cGvHD).

In a stepwise regression analysis, cGvHD significantly predicted (*β*= 165, *t*(184)= 2.14, *p*= .034) the sum score of comorbidities (PTMI), while age and prior acute GvHD had no predictive effects on the PTMI. According to this model, a small but significant proportion of variance (*R*²= .02, *F*(1,184)= 4.59, *p*= .034) could be explained by the independent variable cGvHD. With regard to the HCT-CI, neither cGvHD, nor prior acute GvHD nor age significantly predicted the sum score of comorbidities detected by the HTC-CI indicating, that effects of cGvHD are not captured by the HCT-CI. Regarding the FCI, cGvHD significantly predicted (*β*= .177, *t*(183)= 2.47, *p*= .015) the quantity of comorbidities captured by the FCI. In contrast to the PTMI, age also significantly predicted (*β*= .167, *t*(183)= 2.33, *p*= .021) the FCI (age related effects on the sum score of comorbidities captured by the FCI), while acute GvHD had no predictive effect. According to this model, a significant proportion of variance, (*R*²= .06, *F* 2,183)= 5.61, *p*= .004), could be explained by the independent variables age and cGvHD.

Concerning the CCI, acute GvHD significantly predicted, (*β*= .173, *t*(183)= 2.40, *p*= .017), the number of comorbidities. In contrast to the PTMI age also significantly predicted (*β*= .148, *t*(183)= 2.06, *p*= .041) the CCI, while cGvHD had no predictive effect. According to this model, a small but significant proportion of variance (*R*²= .05, *F*(2,183)= 5.02, *p*= .008) could be explained.

**Survival analysis**

A total of 24 cGvHD patients (17.6%) of the validation cohort did not survive the follow-up period. Kaplan Meier analysis revealed a higher TRM in patients with cGvHD and low platelet counts at onset of cGvHD compared to patients with cGvHD and platelets > 100G/l (Figure 1) with a median follow up of 3216 days after alloHSCT. This difference approached significance (log rank test:χ²(1) = 5.006, *p*= .025). The cumulative number of comorbidities (<3 vs. >3 or <6 vs. >6 comorbidities) had no significant impact on overall survival and TRM. Weighting of comorbidities as performed within the HCT-CI did not improve the association with TRM.
